# A Prognostic Model Based on Cisplatin-Resistance Related Genes in Oral Squamous Cell Carcinoma

**DOI:** 10.3290/j.ohpd.b4836127

**Published:** 2024-01-15

**Authors:** Rong Lu, Qian Yang, Siyu Liu, Lu Sun

**Affiliations:** a Attending Physician, Department of Clinical Laboratory, Shengli Oilfield Central Hospital, Dongying, Shandong, China. Designed the study and conducted the experiment, read and agreed to the publication of this study.; b Attending Physician, Department of Stomatology, Shengli Oilfield Central Hospital, Dongying, Shandong, China. Designed the study, conducted the experiment, read and agreed to the publication of this study.; c Resident Doctor, Department of Stomatology, Shengli Oilfield Central Hospital, Dongying, Shandong, China. Analysed the data, read and agreed to the publication of this study.; d Attending Physician, Department of Stomatology, Shengli Oilfield Central Hospital, Dongying, Shandong, China. Wrote the manuscript, read and agreed to the publication of this study.; + The first two authors contributed equally.

**Keywords:** cisplatin, gene, oral squamous cell carcinoma, prognosis, resistance

## Abstract

**Purpose:**

To screen for the cisplatin resistance-related prognostic signature in oral squamous cell carcinoma (OSCC) and assess its correlation with the immune microenvironment.

**Materials and Methods:**

The gene expression data associated with OSCC and cisplatin-resistance were downloaded from TCGA and GEO databases. Cisplatin-resistant genes were selected through taking the intersection of differentially expressed genes (DEGs) between tumor and control groups as well as between cisplatin-resistant samples and parental samples. Then, prognosis-related cisplatin-resistant genes were further selected by univariate Cox regression and LASSO regression analyses to construct a survival prognosis model. A GSEA (gene set enrichment analysis) between two risk groups was conducted with the MSigDB v7.1 database. Finally, the immune landscape of the sample was studied using CIBERSORT. The IC50 values of 57 drugs were predicted using pRRophetic 0.5.

**Results:**

A total 230 candidate genes were obtained. Then 7 drug-resistant genes were selected for prognostic risk-score (RS) signature construction using LASSO regression analysis, including *STC2, TBC1D2, ADM, NDRG1, OLR1, PDGFA* and *ANO1*. RS was an independent prognostic factor. Additionally, a nomogram model was established to predict the 1-, 2-, and 3-year survival rates of samples. The GSEA identified several differential pathways between two risk groups, such as EMT, hypoxia, and oxidative phosphorylation. Fifteen immune cells were statistically significantly different in infiltration level between the two groups, such as macrophages M2, and resting NK cells. A total of 57 drugs had statistically significantly different IC50 values between two risk groups.

**Conclusion:**

These model genes and immune cells may play a role in predicting the prognosis and chemoresistance in OSCC.

Head and neck squamous cell carcinoma (HNSC) is a significant public health problem around the world.^42^ Among HNSC cases, oral squamous cell carcinoma (OSCC) accounts for 30%.^42^ Smoking, drinking alcohol, and chewing areca nut are the main risk factors for OSCC.^3^^,^^21^ 377,713 new cases and 177,757 deaths from OSCC were reported worldwide in 2020.^35^ With the increase in the morbidity and mortality, OSCC presents a 5-year survival rate of<50%.^28^ Importantly, OSCC has a high recurrence rate of 18% to 76%, even in patients who received standard treatments. Patients with recurrent and metastatic disease have a particularly poor prognosis.^40^ Hence, exploring effective prognostic models is essential for predicting the survival of OSCC patients and guiding clinicians throuh the treatment process.

Recently, there have been some prognostic gene-signatures identified in OSCC,^2^^,^^40^ but the predictive value needs further validation. Currently, for the treatment of OSCC, chemotherapy is an effective method, especially for patients with advanced cancers.^7^ Chemotherapy can reduce the distant metastasis rates and preserve organ function, with or without combined local/regional therapy.^25^ Cisplatin is a potent chemotherapeutic agent, and acts cytotoxically by forming intra-strand DNA cross-linked adducts.^26^ However, in some patients, the therapeutic effects of cisplatin-induced DNA damage resulting in apoptosis may be diminished, and consequent drug resistance is a main limitation of this chemotherapy. Cisplatin-resistance has led to a worse prognosis for patients with OSCC.^30^^,^^31^ Therefore, it is critical to identify cisplatin-resistance biomarkers in order to predict the therapeutic response and prognosis of OSCC patients. However, to the authors’ best knowledge, a cisplatin resistance-related prognostic model has not yet been reported for OSCC.

This study aimed to screen for a cisplatin-resistance–related prognostic signature in OSCC and assessed its correlation with the immune microenvironment. The gene expression data associated with OSCC and cisplatin resistance were downloaded from the TCGA and GEO databases. Cisplatin-resistant genes were selected through taking the intersection of differentially expressed genes (DEGs) between tumor and control groups as well as between cisplatin-resistant and parental samples. Then, prognosis-related cisplatin-resistant genes were further selected by univariate Cox regression and LASSO regression analyses to a construct survival prognosis model. Finally, the immune landscape of the sample was studied.

## MATERIALS AND METHODS

### Acquisition and Screening of Expression Profile Data

Gene expression RNAseq sequencing data (log2 (fpkm+1)) of HNSC were downloaded from the UCSC Xene platform.^15^ Samples from tongue, mouth, gum, lip, cheek mucosa and palate were selected. Then, samples with number “-01A” were screened as an OSCC sample for analysis, while the sample with number “-11A” served as a normal control sample. A total of 255 cancer and 19 normal control samples were included, and the 255 OSCCs provided both survival and clinical information. According to the downloaded gene annotation file GENCODE V22, the genes with annotation information as “protein_coding” were reserved for analysis.

The OSCC data set GSE42743 was downloaded from the NCBI GEO database. In all, 71 OSCC samples with survival information were selected from this dataset. Additionally, GSE111585 and GSE115119 were also downloaded. GSE111585 contains three cisplatin-resistant tongue squamous-cell carcinoma (TSCC) cell lines and three parental SCC9 cell lines. GSE115119 contains two cisplatin-resistant TSCC cell lines and two parental CAL-27 cell lines. Furthermore, the preprocessed, standardised and log2-transformed probe expression matrixes were downloaded. The annotation file of the platform was downloaded for one-to-one matching of probe number and gene symbol.

### Screening of Cisplatin Resistance Genes

Based on the gene expression matrices of GSE111585 and GSE115119, differential gene analysis was carried out on cisplatin-resistant vs parental cell lines using the classical Bayesian method in limma 3.10.3.^34^ BH correction was adopted to obtain the adjusted p-value of DEGs. The adjusted p<0.05 and |logFC|>1 were the thresholds. After that, the DEGs from two datasets were compared, and the genes with consistent up- and down-regulation were screened as cisplatin-resistant genes.

### Screening of Candidate Genes

Based on the gene expression matrices of TCGA-OSCC, differential gene analysis of tumor vs normal was performed as above. The thresholds of DEGs were set as follows: adjusted p<0.05 and |logFC|>0.5. The obtained DEGs were then intersected as the candidate genes.

### Prognosis-related Differentially Expressed Resistant Genes (DERs) Screening

Based on the expression levels of DERs in all OSCC samples, combined with the survival and prognosis information of the samples, univariate Cox regression analysis was carried out using R3.6.1 survival 2.41-1^41^ to screen telomerase genes related to the overall survival prognosis. p<0.05 was used as the threshold.

### Construction of the Prognostic Signature of DERs

On the basis of the DERs statistically significantly related to survival prognosis obtained in the previous step, combined with the survival prognosis information of the training set TCGA-OSCC samples, a LASSO Cox regression model38 in R3.6.1 glmnet 2.0-1811 was used to further screen prognosis-related resistance genes. After that, the following Risk Score (RS) model was constructed:

$$\text{RS}={\text{Σβ}}_{\text{gene}}\times {\text{Exp}}_{\text{gene}}$$

where ${\text{β}}_{\text{gene}}$ represents the LASSO Cox regression coefficient of each gene in the differential resistance gene combination, and Expgene represents the expression level of each gene in the differential resistance gene in each sample.

Further, in order to verify the the model, the RS values of each OSCC sample in the training (TCGA-OSCC) and verification (GSE42743) sets were calculated by using the same regression coefficient. Then, all OSCC samples from the training and verification sets were divided into high- and low-risk sample groups, respectively, according to the RS median. The association between the risk groups and actual survival prognosis was assessed using the Kaplan-Meier (KM) survival curve method 2.41-1. At the same time, combined with the survival information of the samples, the 1, 2, and 3-year survival-prediction receiver operator characteristic (ROC) curves of the model were drawn in the training and verification sets.

### Correlation Analysis Between RS and Clinicopathological Characteristics

In the training set of TCGA-OSCC samples, the Wilcoxon test was applied to calculate the significant difference of RS between the groups according to the clinical characteristics, and a box-and-whisker plot was drawn. Additionally, KM survival curves of RS under different clinical factor grouping were drawn and the logRank test was used to calculate p-values.

### Independent Analysis of the Prognostic Model and Establishment of the Normgram

To determine whether the RS model can serve as an independent prognostic factor, univariate Cox regression analysis was conducted for age, gender, neoplasm histologic grade, tumor stage, pathologic N, pathologic T, and RS. Variables with p<0.05 were further subjected to multivariate Cox regression analysis to further screen out variables with p<0.05 as independent prognostic factors. A nomogram was drawn with these independent prognostic factors. Meanwhile, the calibration curve and the 1, 2, and 3-year survival-prediction ROC curves were drawn.

### GSEA of HALLMARK Gene Sets Between Risk Groups

With MSigDB v7.1^23^ database h.all.v7.4.symbols.gmt as the enrichment background, a GSEA was performed using R clusterProfiler^45^ on the basis of the expression values of all genes in TCGA-OSCC samples and combined with the risk grouping information of the samples. Adjusted p<0.05 was considered a statistically significant enrichment result.

### Associations Between Risk Groupings with Immune Microenvironments

In order to observe the differences of immune microenvironment between risk groups, the CIBERSORT^19^ algorithm was used to calculate the proportion of 22 kinds of immune cells according to the gene expression levels in TCGA-OSCC samples. Furthermore, combined with the risk grouping of samples, the Wilcoxon test was used to calculate the p-value. Then, Spearman’s correlation coefficient and the corresponding statistical significance of the p-value between the model gene and the differential immune cells were calculated, and the correlation heatmap was drawn. The results with correlation coefficient>0.3 and p<0.05 were depicted in a scatter diagram.

### Relationships Between Risk Groups and Immune Checkpoint Gene Expression

The expression levels of immune checkpoint genes (PDCD1 (PD-1), CD274 (PD-L1), IDO1, CTLA4, TIGIT, CD96, PVR and LAG3) were extracted from the TCGA-OSCC dataset. The Wilcoxon test was used to compare the intergroup differences.

### Drug Sensitivity Analysis in High- and Low-risk Groups

To observe the differences of IC50 values of sensitivity to common chemotherapy agents between two risk groups, the pRRophetic algorithm was used to construct a Ridge regression model to predict the IC50 of drugs according to the GDSC cell-line expression profile and the TCGA-OSCC gene expression profile. Here, R package pRRophetic 0.5^13^ was used to predict IC50 values based on the common drugs provided in this package, and the Wilcoxon test was used to calculate statistically significant differences.

### Analysis of Differences Between High- and Low-risk Groups

With the gene expression matrixes of TCGA-OSCC tumor sample, DEGs of high-risk vs. low-risk were identified as above. The thresholds were adjusted p<0.05 and |logFC|>0.5.

Furthermore, GO^1^ (including BP, CC, MF) and KEGG pathway^18^ enrichment analyses were carried out on the DEGs between the two risk groups based on the online tool DAVID.^32^ The number of enriched genes=2 and p<0.05 were set as thresholds. The top 10 items were selected for display.

## RESULTS

### Data Preprocessing and Resistance Genes Screening

**Fig 1:**
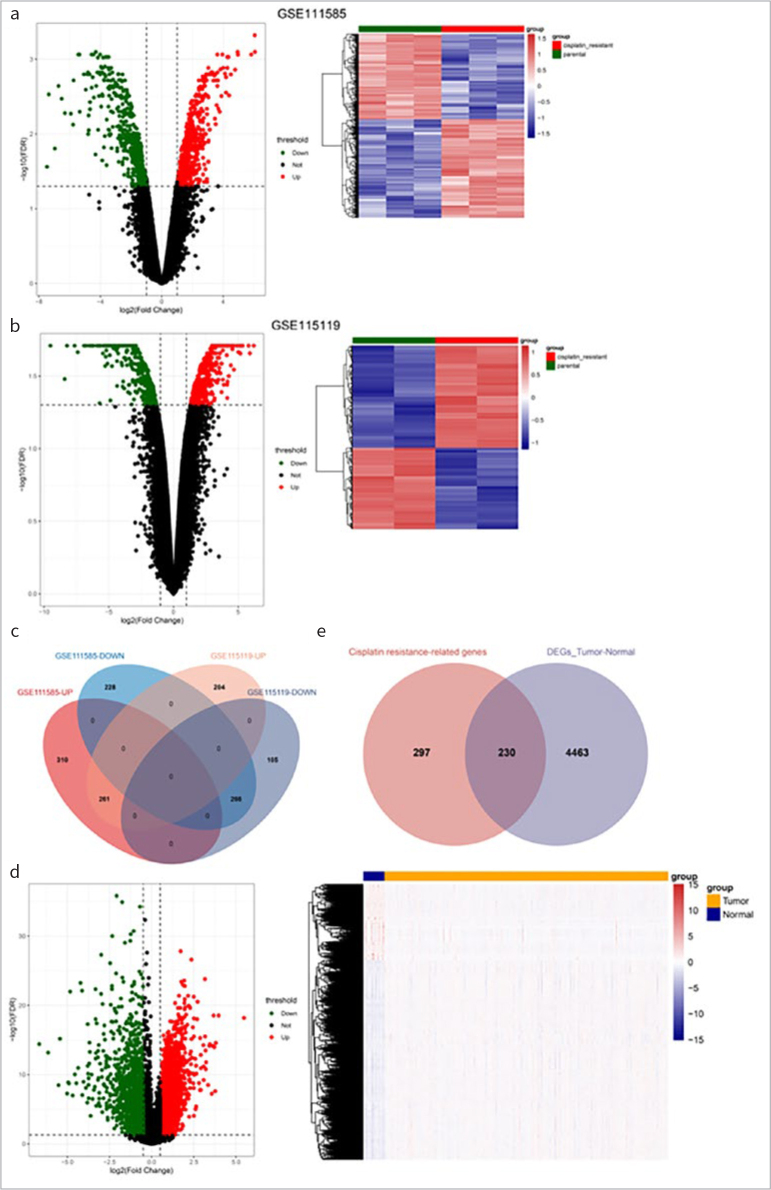
Screening of differentially expressed resistance genes. (a) and (b): Volcanic map and heat map of differentially expressed genes (DEGs) between cisplatin resistant vs. parental in GSE111585 (a) and GSE115119 (b). (c): The intersection Venn diagram of cisplatin-resistance genes. (d): Volcanic map and heat map of DEGs in tumor vs normal. (e): The intersection Venn diagram of DEGs in tumor vs. normal and cisplatin resistant vs parental.

After gene annotation, the expression values of 19,710 genes in 255 cancer samples and 19 normal samples in the TCGA database were finally obtained. Differential analysis of cisplatin-resistant vs parental cell lines identified 571 up- and 494 down-regulated genes in GSE111585, and 465 up-regulated and 371 down-regulated genes in GSE115119. Volcanic maps and heat maps of the two datasets are shown in Figs1a and 1b, respectively. The intersection of up- and down-regulated DEGs in the two datasets was uniformly taken, as shown in Fig1c, and 261 up-regulated and 266 down-regulated drug-resistant genes were finally obtained.

### Screening of Candidate Genes

A total of 4693 DEGs were obtained by differential analysis of tumor vs normal (Fig1d). Then, these DEGs and the above-mentioned drug-resistant genes were intersected, and as shown in Fig5, 230 DERs were obtained (Fig1e).

### Prognostic-related DERs Screening and Prognostic Signature of DERs Construction

**Fig 2:**
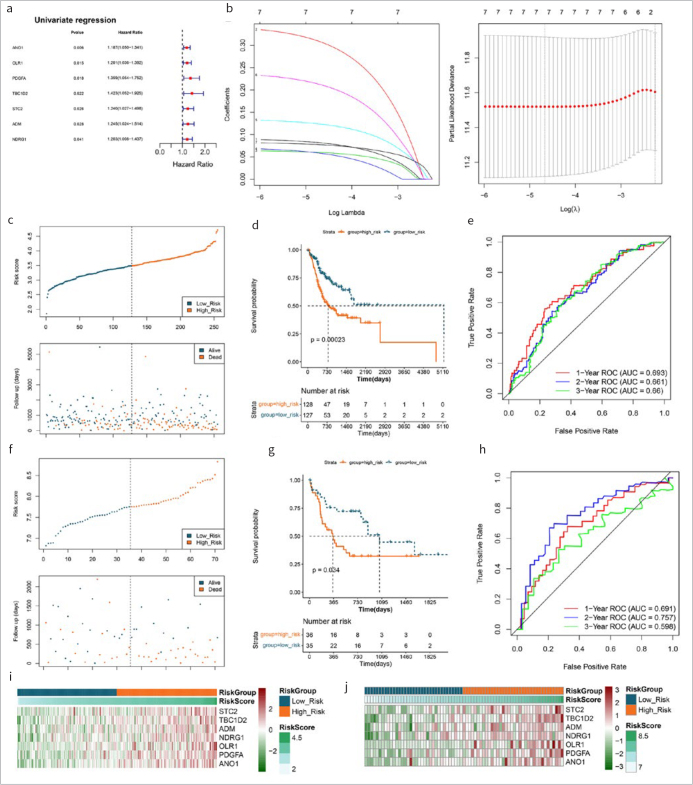
>Prognostic-related DERs screening and prognostic signature of DERs construction. (a): Univariate regression forest maps of 7 prognosis-related differentially expressed resistant genes (DERs). (b): LASSO filter parameter diagram. (c) and (f): RS distribution and survival time state in TCGA and GSE42743. (d) and (g): KM curves related to 8 optimized gene prognostic models in TCGA and GSE42743. Blue and red curves represent low risk and high risk sample groups respectively. (e) and (h): ROC curve curves of 7 optimal gene prognostic models in TCGA and GSE42743. (i) and (j): Expression heat maps of model genes in training set TCGA OSCC (i) and validation set GSE42743 (j).

Based on the DERs, univariate Cox regression was performed, which yielded 7 genes (p<0.05) (Fig2a). The LASSO Cox regression algorithm identified 7 optimized drug-resistant gene combinations for model construction, including *STC2, TBC1D2, ADM, NDRG1, OLR1, PDGFA* and *ANO1* (Fig2b).

According to the LASSO Cox regression coefficients of 7 optimised drug-resistant genes and their expression levels in each sample of the TCGA training set and the GSE42743 validation set, an RS model was constructed. The RS of each sample is shown in Figs2c and 2f. Then, samples from the training and verification sets were grouped into high- and low-risk samples. The association between the risk grouping and actual disease-prognostic information was evaluated using a KM curve. As shown in Figs2d and 2g, the prognosis in the low-risk group was statistically significantly better than that in the high-risk group. The 1-, 2- and 3-year survival prediction ROC curves are shown in Figs2e and 2h. The results indicated a statistically significant correlation between two risk groups predicted by the RS model and actual prognosis. The heatmaps of model genes in training and validation sets revealed that the expression of model genes increased with increasing risk score (Figs2i and 2j).

### Correlation Analysis Between RS and Clinicopathologic Characteristics

**Fig 3:**
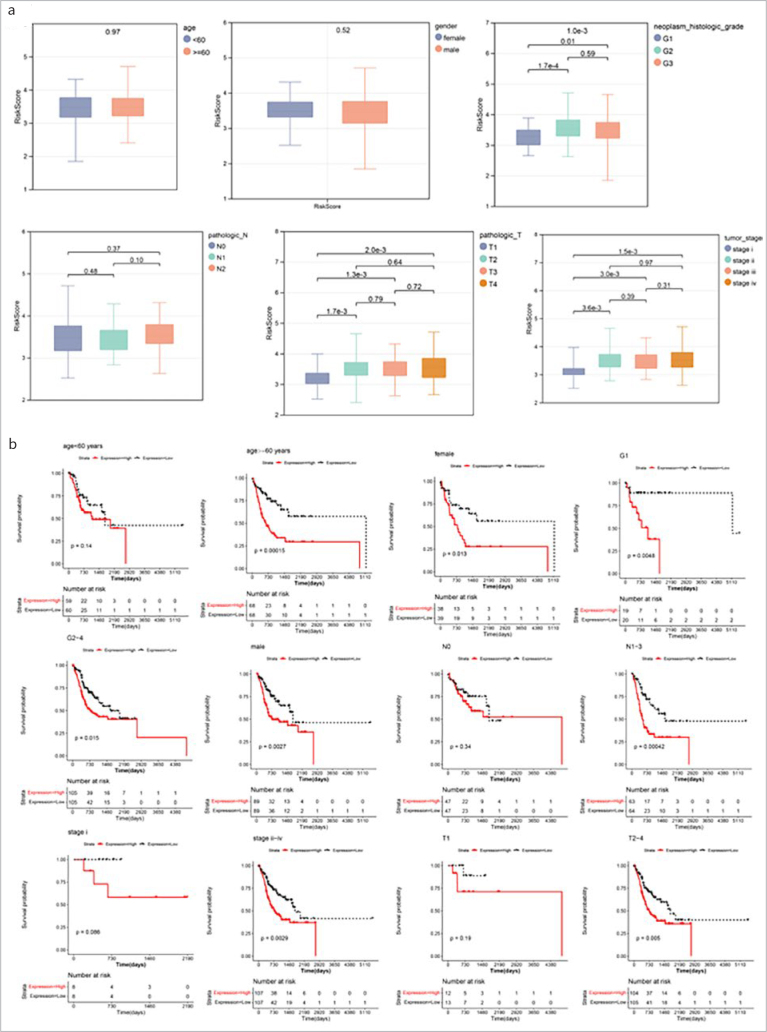
Correlation analysis between RS and clinicopathologic characteristics. (a): Box-and-whisker plots of RS distribution across different clinical groups (top numbers indicate significant p-values). (b): KM curves of RS between different clinical groups (red represents high-risk group, black represents low-risk group).

In the training dataset, statistically significant differences were identified in RS between two risk groups at the T stage, tumor stage and neoplasm_histologic_grade, while other clinical factors did not differ statistically significant between groups (Fig3a). According to different clinical characteristics, KM survival curves of RS under different clinical factor groups were drawn (Fig3b).

### Independent Analysis of Prognostic Model and Normgram Establishment

**Fig 4:**
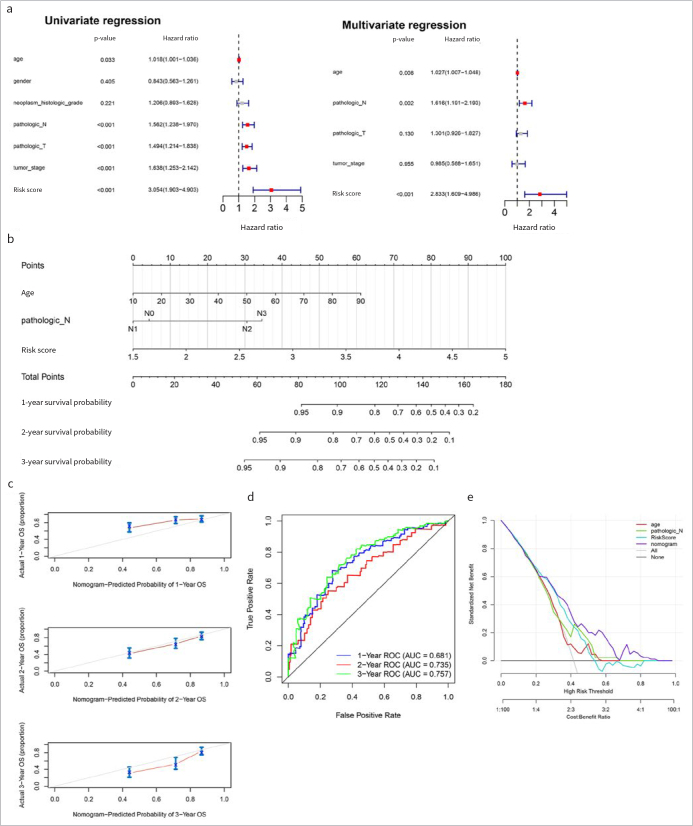
Independent analysis of prognostic model and normgram establishment. (a): Forest maps of clinical factors in univariate and multivariate Cox regression analyses. (b): Independent prognostic factors nomogram of survival prediction model. (c): Calibration curve of 1-, 2-, and 3-year survival predictions with actual survival. (d): The 1-, 2-, 3-years ROC curves of nomogram. (e): Decision curves for gender, pathologic N, stage and RS model.

Following univariate and multivariate Cox regression analyses, age, pathologic_N and RS were considered to be independent prognostic factors (Fig4a), and a nomogram was drawn for these factors (Fig4b). The survival time of the samples was predicted on the basis of the “Total Points” axis in the first line. The predicted 1-, 2-, and 3-year survival rates were in accordance with the actual survival rates (Fig4c). The AUCs of 1-, 2-, and 3-year ROC curves were 0.681, 0.735 and 0.757, respectively (Fig4d). In addition, decision curves for gender, pathologic N, stage and RS model are presented in Fig4e.

**Fig 5:**
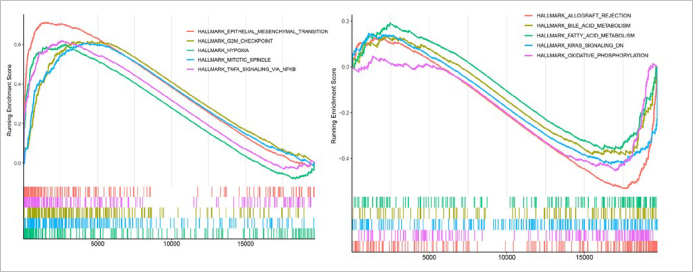
GSEA of HALLMARK gene sets between risk groups. GSEA enrichment results of HALLMARK gene set in high-risk vs low-risk (top 5 up-regulated [left] and down-regulated [right]).

### GSEA Results

In total, 26 up-regulated KEGG signaling pathways and 5 down-regulated pathways were screened. The top 5 up-regulated pathways included epithelial mesenchymal transition (EMT), G2M checkpoint, hypoxia, mitotic spindle, and TNFα signaling via NFκB. The 5 down-regulated pathways were allograft rejection, bile acid metabolism, fatty acid metabolism, KRAS signaling DN, and oxidative phosphorylation (Fig5).

### Associations Between Risk Groupings and Immune Microenvironments

**Fig 6:**
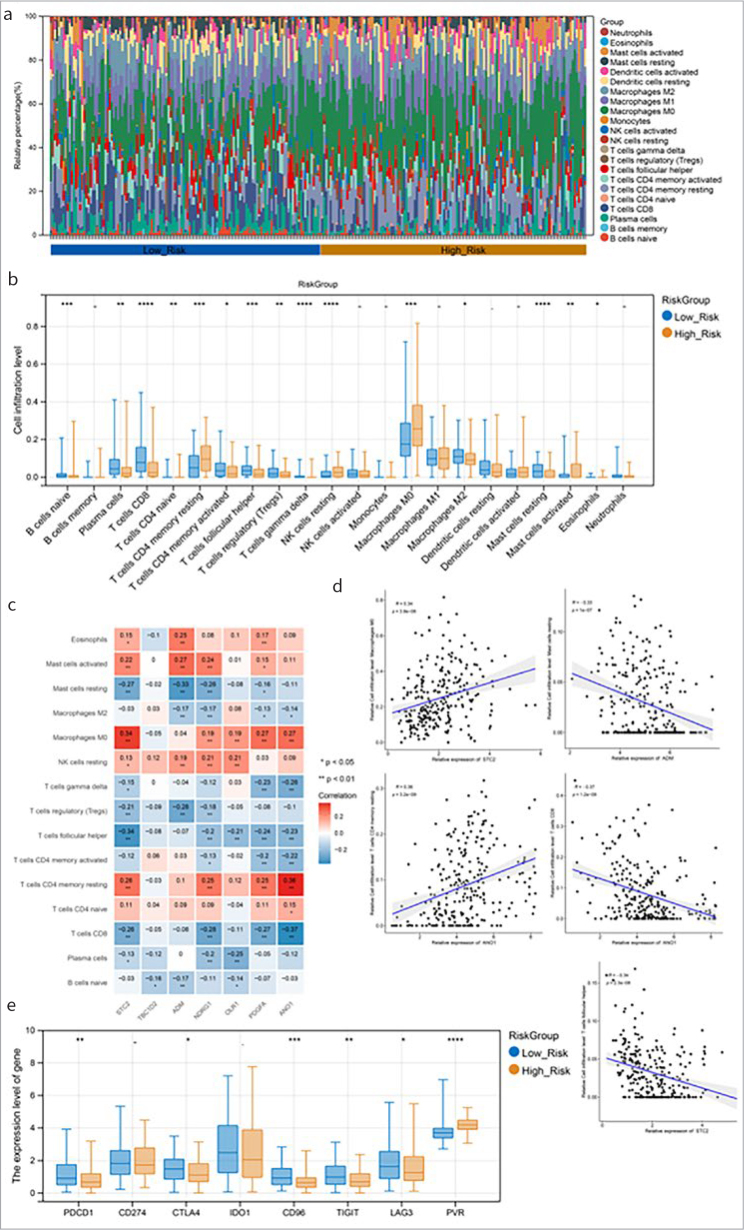
Associations between risk groupings and immune microenvironments. (a): The relative proportions of 22 types of immune cells. (b): Horizontal box-and-whisker plot of immune cell infiltration levels in high- and low-risk groups. (c): Heat maps of correlations between model genes and 15 different immune cells (numbers are correlation coefficients; *p<0.05, **p<0.01, ***p<0.001, **** p<0.0001). (d): The gene-cell correlation scatter plot of correlation coefficient >0.3 and p<0.05. (e): Box-and-whisker plot of immune checkpoint gene expression between high- and low-risk groups.

The relative proportions of 22 kinds of immune cells in the training set were visualised using the accumulation diagram (Fig6a). Fifteen immune cells were differed statistically significantly in terms of infiltration level between the two groups, e.g., naïve B cells, regulatory T cells, macrophages M2, and resting NK cells (Fig6b). Correlation analysis revealed that T cells CD4 memory resting had the highest positive correlation with *ANO1*. T cells CD8 had the lowest positive correlation with *ANO1* (Fig6c). The gene-cell relationship pairs with |correlation coefficient|>0.3 and p-value<0.05 were visualised in the scatter diagram (Fig6d). Moreover, six immune checkpoints (PDCD1, CD96, CTLA4, TIGIT, PVR and LAG3) were differed statistically significantly between the two risk groups (Fig6e).

### Drug Sensitivity Analysis

**Fig S1:**
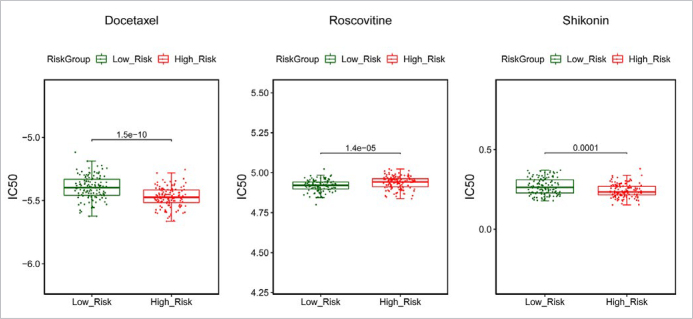
Drug sensitivity analysis. Distribution of IC50 values of docetaxel, roscovitine and shikonin among high- and low-risk groups.

A total of 57 drugs had significant differences in IC50 values between the two risk groups, and three common drugs, including docetaxel, roscovitine and shikonin, are shown in Fig S1.

### Analysis of Differences Between High and Low Risk Groups

**Fig 7:**
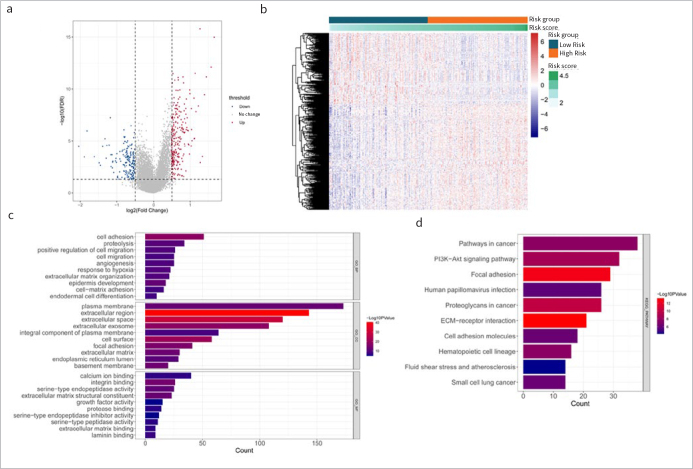
Analysis of differences between high and low risk groups. (a) and (b): Volcano map and heat map of differentially expressed genes in high risk vs low risk. (c) and (d): The top 10 bars of GO enrichment results and KEGG pathway enrichment results.

A total of 254 up- and 170 down-regulated genes were obtained by differential analysis in high-risk vs low-risk groups (Figs7a and 7b). GO and KEGG enrichment analyses were conducted for the above DEGs. A total of 224 BP, 69 CC, 65 MF and 61 KEGG pathways, such as focal adhesion, PI3K-Akt signaling pathway, and ECM-receptor interaction were enriched. The top 10 results of each category are shown in Figs7c and 7d.

## DISCUSSION

Cisplatin has a broader range of drug resistance during the anti-tumor process.^8^ This study was designed to identify cisplatin-resistant biomarkers to predict the prognosis of patients with OSCC. Four cohort datasets from different groups were analysed in this study. Preliminarily, 230 DERs were identified. Finally, 7 drug-resistant genes were selected for prognostic RS signature construction using LASSO regression analysis, including *STC2, TBC1D2, ADM, NDRG1, OLR1, PDGFA* and *ANO1*. RS was an independent prognostic factor. GSEA identified several differential pathways between two risk groups, such as EMT, hypoxia, and oxidative phosphorylation. Fifteen statistically significantly different immune cells were identified between the two groups.

Seven drug-resistant genes were finally selected for prognostic signature construction, i.e., *STC2, TBC1D2, ADM, NDRG1,* and *OLR1*. Most of these genes have been demonstrated to be related to the prognosis of OSCC or other cancers. For instance, *STC2* (stanniocalcin 2) encodes a glycoprotein hormone involved in phosphate and calcium homeostasis.^20^ Additionally, up-regulation of this gene is related to apoptosis, proliferation, invasion and EMT in tumors.^27^ Recently, *STC2* was reported to contribute to the aggressiveness of OSCC and is a prognostic marker for this cancer.^10^ High expression of *TBC1D2* (TBC1 domain family member 2) was proven to be related to the poor prognosis of patients with ovarian cancer.^37^ *ADM* (adrenomedullin peptide) is a proto-oncogene that plays multiple roles in cancers.^46^ ADM can induce the expression of genes associated with lymphangiogenesis and angiogenesis.^24^ NDRG1 (N-Myc downstream regulated 1) is a tumor metastasis suppressor, and overexpression of NDRG1 is associated with lower metastatic and invasive potentials, as well as increased susceptibility to chemotherapeutic agents.^4^^,^^6^ OLR1 is a stimulator of EMT and can promote migration and metastasis of human cancers, including HNSC.^39^^,^^44^ Overall, our study further suggested the prognostic implications of these biomarkers in OSCC.

GSEA identified several differential pathways between two risk groups, such as EMT, hypoxia, and oxidative phosphorylation pathways. EMT is a process in which epithelial cells lose their intercellular adhesion and gain properties of invasiveness and migration, which is a prerequisite for metastasis.^29^ Importantly, EMT is implicated in the resistance of tumor cells to radio- and chemotherapy.^14^^,^^16^ Hypoxia stress is the signature pathological feature of tumor cells in most patients with cancers.^17^ There is increasing evidence that a hypoxic microenvironment is a major factor in tumor insensitivity to cisplatin treatment.^9^ Oxidative phosphorylation contributes to cancer progression, and exacerbated oxidative phosphorylation dependency is characteristic of cancer stem cells, as well as primary or acquired resistance against chemotherapy.^33^ Inhibition of oxidative phosphorylation could statistically significantly reduce cisplatin resistance.^43^ Therefore, the different risk of cisplatin resistance in two groups may be due to the differences of EMT, hypoxia and oxidative phosphorylation in OSCC.

A recent study demonstrated that the tumor microenvironment plays a key role in the development of cisplatin resistance.^5^ Thus, we evaluated the proportion of immune cell types and compared their differences in the distribution between the two risk groups. Fifteen immune cell types were obtained, such as macrophages M2, and resting NK cells. Macrophages have a high infiltrated level among the immune cells in the tumor microenvironment, and emerging evidence shows that macrophages contribute to chemoresistance.^22^ NK cells, a key player in the innate immune system, have a critical role in tumor immune monitoring and prevention of metastasis progression. Human tumors with more NK-cell infiltration are associated with improved prognosis and reduced tumor recurrence.^36^ Decreased infiltration of NK cells into tumor tissue may be a predictor of chemotherapy failure in breast cancer.^12^ Thus, our results further indicate the key role of the immune-cell proportions in predicting the prognosis of and chemoresistance in OSCC.

## CONCLUSION

Seven cisplatin-resistance related genes were finally screened to construct an RS model. These model genes may be associated with cisplatin-resistance in OSCC via EMT, hypoxia and oxidative phosphorylation pathways. Furthermore, the proportion of immune cells may play a role in predicting prognosis and chemoresistance in cases of OSCC.
